# Iodine Treatment, Tendinous Synovial Dilatations

**Published:** 1902-06

**Authors:** 


Iodine Treatment, Tendinous Synovial Dilatations. Labat has had much success in such synovial distentions with the following solution: Tincture of iodine, 100 g.; boiling water, 200 g.; iodide of potassium, 6 g. He prefers the animal cast, and makes two successive injections in the usual manner. The results from this treatment of thoroughpin, cysts of the back, windgalls and distentions around the knee have been much better than from blistering and cauterization.

In thirty-five cases of distentions and cysts of the back, four of the knee and fourteen windgalls, the results were satisfactory, excepting after the first injection. The resolution requires from two to five months. Cadéac also recently has recommended this treatment as almost infallible.

